# Arthrose due au *genu varum*: traitement par osteotomie tibiale de valgisation

**DOI:** 10.11604/pamj.2014.19.171.4297

**Published:** 2014-10-20

**Authors:** Abdou Kadri Moussa, Loubet Unyendje Lukulunga, Mustapha Mahfoud, Ahmed El Bardouni, Farid Ismail, Mohamed Kharmaz, Mohammed Saleh Berrada, Moradh El Yaacoubi

**Affiliations:** 1 Service de traumatologie orthopédie CHU Ibn Sina, Rabat, Maroc

**Keywords:** Genu varum, arthrose, ostéotomie, Genu varum, osteoarthritis, osteotomy

## Abstract

Le traitement du genu varum est le plus souvent conservateur (ostéotomie tibiale de valgisation) permettant de corriger le trouble architectural afin de rétablir l'axe physiologique du membre inférieur. Le but de l’étude était d’évaluer les résultats du traitement et comparer à ceux de la littérature. Il s'est agi d'une étude rétrospective portant sur des patients présentant un genu varum qui s'est déroulée dans le Service de Chirurgie Orthopédique et Traumatologie de CHU Ibn SINA de RABAT, sur une période de 9 ans (2000 au 31 Décembre 2008). Nous avons inclus dans notre étude: les patients qui avaient un genu-varum clinique avec examen radiographique standard ainsi qu'un pangonogramme; traités par différents procédés d'ostéotomie tibiale de valgisation; avec un suivis d’ au moins deux ans. Nos critères d’évaluation ont été appréciés selon le score HSS. Nous avons colligé 115 cas de genu-varum par ostéotomie de valgisation. L’âge de nos patients variait entre 40 et 69 ans, avec une moyenne de 53 ans. Le pic de fréquence se situait entre 52et 63 ans. Le sexe féminin prédominait avec 87 cas (75,6%) avec un sex ratio 3,1. Un Indice de masse corporelle supérieur à 30 a été noté dans 44 cas (38%). Quant aux antécédents chirurgicaux,18 patients de la série (soit 14%) ont été opérés pour le genu varum d'un autre genou. Le délai de consultation a varié entre 4 mois à 6 ans, avec une moyenne de 2 ans. La douleur était le principal motif de consultation et était de siège médial dans 70% des cas et bicompartimental dans 30% cas. Il s'agissait d'une douleur mécanique dans 76% des cas, mixte 21% des cas et inflammatoire 4% des cas. La déformation du genou appréciée par l’écart intercondylien a été en moyenne de 8,7 cm avec des extrêmes de 3 cm et 33cm. Le bilan de l'imagerie médicale reposait essentiellement sur les radiographies standards du genou de face et de profil, ainsi que la goniométrie. Ces clichés nous ont permis de classer l'arthrose du genou selon Ahlbäck. 72,5% des patients, présentaient une arthrose débutante. Le pangonogramme a été réalisé pour mesurer la déviation axiale du génu varum. La déviation angulaire: HKA (angle entre le centre de la tête fémorale et le milieu de la cheville) préopératoire a varié entre 163° et 176°, soit une moyenne de 175,46°. Une correction moyenne de déviation de 11,3° a été réalisée avec des extrêmes de 7 à 19°. Cet angle de correction (DAC) qui variait de 7 à 19° a été supérieur à 15° dans 57,39%s inférieur à 15° chez 38%. 27,4% des patients avaient une déviation angulaire importante avec une arthrose avancée. Après un bilan préopératoire et une planification opératoire 73% des patients ont été opérés sous anesthésie loco-régionale. Pour l'ostéotomie tibiale de fermeture, la voie d'abord a été la voie de Gernez antérolatérale, utilisée chez 56 cas (48,6%), l'ostéotomie tibiale d'ouverture (la voie d'abord était Gernez antéro-médiale) effectuée dans 20 cas (17,3%);et l'ostéotomie curviplane, a été réalisée par une voie d'abord longitudinale médiane dans 39%. Les ostéosynthèses ont été réalisées dans 51 cas (44,3%) par les agrafes de Blount, dans 54 cas (46,9%) par la plaque en T ou en L et dans 11 cas par une plaque en col de cygne. En per-opératoire nous avons enregistré deux (02) cas de fractures du plateau tibial médial, en post-opératoire on a eu 1 cas d'infection superficielle et comme complications tardives une raideur du genou (18,2%) et 3,4% de pseudarthrose, une récidive du génu varum dans 19,1% (n = 22) après 3 années de recul. Les résultats du traitement ont été bons dans 78,4% selon le score HSS. L'OTV sur genu varum a été réalisé chez des patients relativement âgés avec la prédominance du sexe féminin. Les techniques utilisées dépendaient de la préférence du chirurgien. Les résultats sont probants malgré la fréquence des récidives. Quelle que soit la technique utilisée les résultats sont satisfaisants, et malgré l’âge élevé l'OTV pourrait être une alternative intéressante et toujours d'actualité dans nos pays où la population est à majorité analphabète avec un niveau socioéconomique bas.

## Introduction

Le genu varum est la déviation axiale la plus fréquente du genou [1]. La morphologie du membre inferieur en est souvent la principale cause. Tout le poids du corps passe par la partie du genou entrainant une usure prématurée du cartilage et du ménisque à l'origine de l'arthrose fémoro-tibiale débutante du genou chez le sujet de moins de 60 ans [2–4]. Ces lésions anatomiques vont occasionner des douleurs au niveau du genou ainsi qu'un enraidissement et une gêne à la marche nécessitant l'utilisation d'anti inflammatoires non stéroïdiens (AINS) et antalgiques.

L’évolution naturelle se fait vers l'usure complète du cartilage, une gêne de plus en plus et une marche de plus en plus difficile avec apparition en plus des lésions ligamentaires aggravant singulièrement la déformation et compliquant donc le traitement chirurgical [5, 6]. Ce traitement est conservateur (OTV: ostéotomie tibiale de valgisation) permettant de corriger ce trouble architectural afin de rétablir l'axe physiologique du membre inferieur [7].

## Méthodes

Il s'est agi d'une étude rétrospective portant sur des patients présentant un genu varum qui s'est déroulée dans le Service de Chirurgie Orthopédique et Traumatologie de CHU Ibn SINA de RABAT, sur une période de 9 ans (de janvier 2000 au 31 Décembre 2008). Nous avons inclus dans notre étude: les patients qui avaient un genu-varum clinique avec examen radiographique standard (les patients ont été classés selon la classification d'AHLBACK (Tableau 1), pangonogramme a été réalisé pour préciser la déviation angulaire; nos patients ont été traités par différents procédés d'ostéotomie tibiale de valgisation et suivis pendant au moins deux ans. Les critères de non inclusion ont été porté sur les patients présentant un genu-varum avec atteinte globale, lésions ligamentaires graves associées, dossier inexploitable.

**Tableau 1:** Classification selon Ahlbäck

Stade	Articulation
I	Pincement articulaire ≤50%
II	Entre 50 et 100%
III	100%
IV	Pincement avec usure osseuse
V	Usure osseuse avec dé-coaptation fémoro-tibiale latérale

Nos critères d’évaluation ont été appréciés selon le score HSS [8]: scores fonctionnels et échelles de qualité de vie.

**Scores fonctionnels:** HSS (The Hospital for Special Surgery Knee Rating Form); Échelle générique: douleur (/30); fonction (/22); amplitude de mouvement (/18); force musculaire (/10); flessum (/10); instabilité (/10).

**Score global:** (/100 = meilleur score); Score final: excellent (85-100); bon (70-84); moins bon (60-69); mauvais (< 60).

La saisie et l'analyse des données ont été faites à l'aide de logiciel word et SPSS Stastic17.0 avec un seuil de signification p

## Résultats

Lors de la réalisation de cette étude nous avons été heurtés à quelques difficultés: l'exploitation des dossiers, suivi régulier des patients, insuffisance de suivi (recul de moins de5 ans pour certains patients), coût élevé des explorations par rapport au niveau socioéconomique.

Nous avons colligé 115 cas de genu-varum par ostéotomie de valgisation. L’âge de nos patients variait entre 40 et 69 ans, avec une moyenne de 53 ans. Le pic de fréquence se situait entre 52et 63 ans. Le sexe féminin prédominait avec 87 cas (75,6%). Le sex-ratio était de 3,1 en faveur des femmes. L'obésité (avec un IMC supérieure à 30) a été notée dans 44 cas, représentant 38% de la série (37 femmes et 7 hommes). 33 patients (soit 28%) ont présenté dans leurs antécédents un traumatisme autour du genou arthrosique. Quant aux antécédents chirurgicaux,18 patients de la série (soit 14%) ont été opérés pour le genu varum d'un autre genou

Parmi les tares associées nous avons enregistré 12 cas d'HTA, 8 cas de diabète, 2 cas d'ulcère, 2 cas d'asthme. 20% de nos patients et particulièrement les personnes âgées présentaient une tare associée Le délai de consultation a varié entre 4 mois à 6 ans, avec une moyenne de 2 ans. La douleur était le principal motif de consultation et existait chez tous les malades. Elle était de siège médial dans 70% des cas et bicompartimental dans 30% cas. Il s'agissait d'une douleur mécanique 76% des cas, mixte 21% des cas et inflammatoire 4% des cas. La boiterie a été notée dans 45 cas (47,7%). La déformation du genou appréciée par l’écart intercondylien était en moyenne de 8,7 cm avec des extrêmes de 3 cm et 33cm. Trente trois patients (soit 29%) présentaient une limitation de la mobilité du genou sans qu'elle soit quantifiée par l'examinateur. Sur le plan de la stabilité 20 patients de notre série ont présenté des troubles de stabilité du genou, tels que: blocage (13%), craquement (3%), dérobement (2%), instabilité franche à l'examen avec mouvements de latéralité (2%).

Le bilan de l'imagerie médicale reposait essentiellement sur les radiographies standards du genou de face et de profil qui ont été systématiques pour tous nos patients ainsi que la goniométrie. Ces clichés nous ont permis de classer l'arthrose du genou selon Ahlbäck [9] (Tableau 2) 72,5% présentaient une arthrose débutante, c'es-à-dire stade I ou stade II Le pangonogramme a été réalisé chez tous nos malades pour quantifier la déviation axiale du génu varum. La déviation angulaire: HKA (angle entre le centre de la tête fémorale et le milieu de la cheville) préopératoire a varié entre 163° et 176°, soit une moyenne de 175,46°. On a fait une correction de déviation moyenne de 11,3° avec des extrêmes de 7 à 19°. Cet angle de correction (DAC) qui variait de 7 à 19° a été supérieur à 15° dans 57,39%s inférieur à 15° chez 38%. 27,4% des patients avaient une déviation angulaire importante avec une arthrose avancée (Tableau 3). La déviation angulaire était comprise entre 10° et 15° dans 42,60%, et supérieure à 15° dans 33,91% Après un bilan préopératoire et une planification opératoire 73% des patients ont été opérés sous anesthésie loco-régionale. Le patient était installé en décubitus dorsal avec mise en place d'un garrot à la racine de la cuisse pour avoir un champ opératoire exsangue. La voie d'abord dépendait du type d'ostéotomie envisagée. En cas d'ostéotomie tibiale de fermeture, la voie d'abord a été la voie de Gernez antérolatérale, utilisée chez 56 cas (48,6%). En cas d'ostéotomie tibiale d'ouverture, la voie d'abord était Gernez antéro- médiale effectuée dans 20 cas (17,3%) L'ostéotomie curviplane, a été réalisée une voie d'abord longitudinale médiane dans 39%. L'ostéotomie est sus-tubérositaire et semi-circulaire, placée derrière le tendon patellaire (rotulien). La fixation de nos ostéotomies (ostéosynthèse) a été réalisée dans 51 cas (44,3%) par les agrafes de Blount, dans 54 cas (46,9%) par la plaque en T ou en L et dans 11 cas par une plaque en col de cygne. On a effectué une immobilisation post-opératoire provisoire par une attelle postérieure dans 57% pendant 10 à 15 jours (dans un but antalgique). Nous avons fait une contention par une genouillère plâtrée dans 26% des cas jusqu’à la consolidation de l'ostéotomie (2 à 3 mois). En per-opératoire nous avons enregistré deux (02) cas de fractures du plateau tibial médial. Tous nos patients ont été mis en postopératoire sous traitement antalgique, antibiothérapie prophylactique pendant 48 heures, traitement anticoagulant pour prévenir les complications thromboemboliques.

**Tableau 2:** Répartition des patients selon le stade radiologique

Stade I	50 cas	44,2%
Stade II	32 cas	28,3%
Stade III	18 cas	15,9%
Stade IV	13 cas	11,5%
Stade V	0 cas	

**Tableau 3:** Rapport entre le pincement femoro-tibial et le degré de déviation angulaire

Pincement fémoro-tibial	Nombre de cas	DA < 10°	10° < DA < 15°	DA > 15°
I	50	18	32	0
II	32	9	17	6
III	19	0	0	19
IV	14	0	0	14
Total	115	27	49	39

Nous avons entrepris immédiatement la rééducation (complément thérapeutique indispensable) pour les ostéotomies tibiales hautes d'ouverture médiale (OTHOM). Elle a été retardée jusqu’à 45 jours (ablation de plâtre) pour les ostéotomies tibiales hautes de fermeture latérale(OTHFL). La rééducation isométrique a été immédiate quel que soit le type de l'ostéotomie. La rééducation active et passive n'a été possible immédiatement que pour les malades non plâtrés. Parmi les complications post-opératoires secondaires, on a eu 1 cas d'infection superficielle et comme complications tardives 18,2% de raideur du genou et 3,4% de pseudarthrose, 19,1% récidive du génu varum (n = 22) après 3 années de recul. Les résultats du traitement ont été bons dans 78,4% selon le score HSS (76 points).

## Discussion

L’âge moyen a été de 53 ans. Cette moyenne d’âge est un peu plus élevée par rapport celles de ARGENSON [10]: 42 ans. Un âge inferieur à 50 ans est un facteur de bon pronostic pour la survie d'une OTV. Ceci s'explique par consultation tardive de nos patients et l’éloignement géographique. Le sexe féminin prédominait avec 75,6% alors que Argenson et al [10] ont trouvé une prédominance masculine (61,98%) dans une série 313 cas. X2 = 47,70, p = 0,000. La différence est significative ainsi le sex-ratio était 1, 47 en faveur de l'homme. Ceci est dû au fait que chez nous le genu varum est très fréquent chez les femmes surtout obèses.

En ce qui concerne l’étiologie nous n'avons trouvé aucune cause, ce qui est concordant avec la littérature [11]. Nous avons noté un IMC supérieur à 30 dans 38,26% des cas (37 femmes et 7 hommes). Les antécédents traumatiques ont été notés au niveau du genou siège du varum. Dans la littérature nous n'avons pas eu des données en rapport avec les antécédents traumatiques. Notre délai de consultation a été de 4mois à 6 ans avec une moyenne de 2ans. Ce délai paraît raisonnable et était liée à l'intensité et à tolérance de la douleur. La douleur a constitué le principal motif de consultation (100%) avec 84 cas de type mécanique (76%), ainsi que la boiterie avec 55 cas. Ce qui est le cas dans la plupart des données de la littérature [12].

Nous avons enregistré 82 cas avec un stade I et II de Ahlbäck (arthrose débutante). La déviation angulaire: HKA préopératoire a varié entre 163° et 176° soit une moyenne de 175,46°. La DAG (déviation angulaire globale) a été de 1,30 avec des extrêmes allant de 7à 19°. La déviation angulaire était importante dans notre série supérieure à 10° dans 76,52%, alors que la déviation angulaire est moins grave lorsque le stade de gonarthrose est précoce [13, 14]. L'OTV a été réalisée sous anesthésie loco-régionale chez 75% des patients. La voie d'abord dépendait du type d'ostéotomie à envisager. Le choix de la technique était aléatoire, et laissé à la préférence du chirurgien. Ainsi la voie de Gernez anterolaterale était effectuée chez 56 patients (ostéotomie de fermeture), Gernez antero médiale dans 20 cas et curviplane chez 39 patients. Et l'ostéosynthèse a été réalisée avec agrafes de Blount dans 51 cas, plaque en T ou L dans 51 cas et la lame plaque en col de cygne 11 cas.

L'ostéotomie tibiale de fermeture a été très effectuée dans notre série contrairement à la plupart des séries où l'ostéotomie tibiale d'ouverture est la plus utilisée [15, 16]. Cette préférence pour nous a été de minimiser le risque de pseudarthrose et de morbidité liée au prélèvement de la greffe et ainsi de permettre l'appui précoce. La rééducation, complément thérapeutique indispensable pour récupérer l'amplitude de l'articulation était isométrique immédiatement quelque soit le type d'ostéotomie. Elle était immédiate pour les OTHOM, et retardée de 45 jours (après ablation de plâtre) pour les OTHFL. Cette rééducation était active et passive immédiatement pour les malades n'ayant pas été immobilisés par plâtre.

Nous n'avons enregistré aucune complication immédiate. Quant aux complications secondaires, un cas d'infection superficielle a été noté. Parmi les complications tardives, nous avons eu 21 cas de raideur du genou (soit 18,2%), 4 cas de pseudarthrose (3,4%), et 22 cas de récidive du genu varum(19,1%) et l'aggravation de l'AFTM globale. Segal.P et al [17] ont retrouvé 44 cas de varus sur 135(p = 0,020), donc la différence est significative. Ce taux élevé de récidive pourrait s'expliquer par l'obésité (IMC supérieur à 30) qui était fréquente dans notre série, mais aussi les antécédents traumatiques sur le genou concerné. Selon Naudie e [8] un indice de masse corporelle supérieur à 25 est un facteur de mauvais pronostic.. Cette raideur est due au délai d'immobilisation assez long avec retard et insuffisance de kinésithérapie.

3,4% de pseudarthrose dans notre série, F. Dubiana et al [5] ont trouvé 3,8% au cours de leur étude(X2= 0,0133 et p = 0,90); la différence est non significative. Malgré ce taux élevé de récidive ainsi que les tares associées, les résultats fonctionnels sont globalement satisfaisants selon les scores HSS avec 78,4% de bons résultats comparativement aux données de la littérature [18, 19]. Lootvoet et Insall retrouvent respectivement 84% et 85% de bons résultats avec un recul de 5 à 10 ans; p = 0,046. La différence est significative. Cette différence s'expliquerait par l'importance du genu varum associé à un stade avancé d'arthrose.

## Conclusion

L'OTV sur genu varum a été réalisé chez des patients relativement âgés avec la prédominance du sexe féminin. Les techniques utilisées dépendaient de la préférence du chirurgien. Les résultats sont probants malgré la fréquence des récidives. Quelle que soit la technique utilisée les résultats sont satisfaisants, et malgré l’âge élevé l'OTV pourrait être une alternative intéressante et toujours d'actualité dans nos pays où la population est à majorité analphabète avec un niveau socioéconomique bas.
